# Determination of Multi‐Steroid Profiles of Cats With Hyperaldosteronism or Other Diseases: A Retrospective Study

**DOI:** 10.1111/jvim.70209

**Published:** 2025-08-14

**Authors:** Alice H. Watson, Lorna C. Gilligan, Angela E. Taylor, Wiebke Arlt, Harriet M. Syme

**Affiliations:** ^1^ Royal Veterinary College London UK; ^2^ Queen Mary, University of London London UK; ^3^ Institute of Metabolism and Systems Research, University of Birmingham Birmingham UK; ^4^ Medical Research Council Laboratory of Medical Sciences London UK; ^5^ Institute of Clinical Sciences, Imperial College London London UK

**Keywords:** feline, primary hyperaldosteronism, steroid metabolome, steroidomics

## Abstract

**Background:**

Multi‐steroid profiles are proven to help with the diagnostic approach to adrenal disorders in humans.

**Objectives:**

Compare liquid chromatography tandem mass spectrometry (LC–MS/MS) and radioimmunoassay (RIA) measurement of aldosterone in feline blood samples. Evaluate multi‐steroid profiles in cats with hyperaldosteronism.

**Animals:**

Client‐owned cats with low/normal aldosterone by RIA (low aldo, *n* = 15), high aldosterone by RIA (high aldo, *n* = 6) and overt primary hyperaldosteronism (PHA, *n* = 6).

**Methods:**

Exploratory retrospective case–control study. Feline blood multi‐steroid profiles were analyzed with LC–MS/MS and compared with results from a commercially available RIA in widespread clinical use.

**Results:**

Aldosterone measurement by LC–MS/MS and RIA was strongly correlated (*r* = 0.93, *p* < 0.001) but showed a bias of 56 (95% CI: 104–7) pmol/L, with RIA giving higher values. Creatinine concentration was not significantly associated with the discrepancy between methods (*p* = 0.95). Cats with PHA had significantly lower serum concentrations of cortisol and glucocorticoid precursors (17‐hydroxyprogesterone, 11‐deoxycortisol), testosterone, and significantly higher dihydrotestosterone and 5α‐dihydroprogesterone concentrations than the low aldo group. Pregnenolone concentrations were significantly lower in the PHA group than in the high aldo group.

**Conclusion and Clinical Importance:**

Quantification of aldosterone by either LC–MS/MS or RIA is adequate for PHA diagnosis in the clinical setting, with appropriate method‐specific reference intervals. Cats with PHA had different concentrations of some steroids compared with the control groups, but overt steroid excess (other than aldosterone) was not observed.

**Abbreviations**
CKD: chronic kidney disease
DHEA: dehydroepiandrosterone
high aldo: high aldosterone by RIA
LC–MS/MS: liquid chromatography–tandem mass spectrometry
low aldo: low/normal aldosterone by RIA
PHA: primary hyperaldosteronism
RIA: radioimmunoassay

## 1 Introduction

Hyperaldosteronism is an endocrine condition whereby excessive aldosterone is secreted by the adrenal gland; hyperaldosteronism is classified as primary or secondary. Primary hyperaldosteronism (PHA) involves excessive aldosterone secretion that is independent of normal physiological regulation by the renin‐angiotensin system, with suppression of renin activity due to the resulting extracellular volume expansion [1, 2]. By contrast, secondary hyperaldosteronism arises when activation of the renin‐angiotensin system results in high renin activity; this stimulates aldosterone secretion. PHA has been identified in cats as a result of unilateral disease (either benign or malignant adrenal neoplasia) [3] or bilateral disease (bilateral adrenal hyperplasia or tumors) [3, 4].

Immunoassays are commonly used to measure aldosterone concentrations in feline clinical samples. Immunoassays are considered less reliable than mass spectrometry because antibody cross‐reactivity and matrix effects often lower specificity [5]. Antibody binding kinetics can limit immunoassay sensitivity [5], especially where other steroids are present in excess and can compete with antibody binding. Mass spectrometry‐based methods are therefore often considered preferable due to higher specificity [5], and the possibility for simultaneous quantification of multiple steroid hormones [6]. Due to steroid hormones having similar structures and molecular weights resulting in similar mass to charge ratios (*m*/*z*), steroids require separation to facilitate differential detection. This can be achieved with liquid chromatography preceding tandem mass spectrometry, facilitating the quantification of multiple steroids in a single low‐volume sample [6]. Targeted steroid metabolome analysis in humans has identified signature steroid profiles associated with disorders of steroid biosynthesis and metabolism, and provided insights into the pathophysiology of adrenal disease [7]. Steroid profiles have not been reported in the peer‐reviewed literature in species with a single CYP11B enzyme for both aldosterone and cortisol synthesis, such as cats [8].

Renal disease impairs the quantification of aldosterone by immunoassay in human samples due to the accumulation of polar metabolites of aldosterone, which can cross‐react with the antibodies used in immunoassays [9, 10, 11]. PHA usually arises in older cats, and as chronic kidney disease is a common condition within this population, we hypothesized that renal disease can falsely elevate the quantification of aldosterone by immunoassays. Furthermore, multiple steroid excesses, including progesterone [12], cortisol [13], estradiol [14], and corticosterone [15], occur in a subset of cats with hyperaldosteronism using immunoassays.

To date, no studies have directly compared immunoassay and mass spectrometry quantification of aldosterone (and other steroids) in cat blood samples. This study aimed to compare quantification of aldosterone by radioimmunoassay (RIA) and liquid chromatography tandem mass spectrometry (LC–MS/MS) and determine whether azotemic chronic kidney disease is associated with discrepant results for aldosterone quantification by RIA and LC–MS/MS. In addition, in an exploratory study, multi‐steroid profiles of cats with PHA were compared with two groups of cats without overt PHA, subdivided into low‐normal and high aldo groups based on aldosterone concentrations quantified by RIA.

## 2 Materials and Methods

### 2.1 Case Selection

This was a retrospective study using LC–MS/MS on residual serum or plasma samples from cats that [1] had documented overt PHA based on hyperaldosteronemia with otherwise unexplained hypokalemia and a unilateral adrenal mass (PHA group, *n* = 6); or [2] that had previously undergone serum or plasma aldosterone quantification by RIA for another study. These cats were selected from a longitudinal health monitoring program and had not had abdominal imaging. A large number of samples were available; samples for LCMS/MS were selected to cover a range of aldosterone and creatinine concentrations. These were sub‐divided into those that had aldosterone concentrations above the RIA reference interval (high aldo group, *n* = 6) or within or below the reference interval (low aldo group, *n* = 15). Systemic hypertension was defined according to the local clinic protocol in place at the time of the case being managed, and was of sufficient severity to justify the prescribing of amlodipine. Cats were classified with azotemic chronic kidney disease (CKD) if the plasma creatinine was over the upper limit of the laboratory reference interval 177 μmol/L (2.0 mg/dL) with a concurrent urine specific gravity less than 1.035, or if creatinine was over 177 μmol/L on two occasions at least 2 weeks apart. Cats were classified as hyperthyroid if they were receiving medication for hyperthyroidism (thiamazole). Samples were only included in the analysis if the hyperthyroidism was clinically controlled and their total thyroxine was below the upper limit of the laboratory reference interval (55 nmol/L or 4.27 ng/dL) at the time of sampling.

Samples for the low and high aldo group cats were collected from cats seen in two first opinion practices in central London (People's Dispensary for Sick Animals, Bow and Beaumont Sainsbury Animal Hospital, Camden) between 2001 and 2021. All aldosterone measurements from these cats were performed in a single diagnostic reference laboratory (Michigan State University Veterinary Diagnostic Laboratory, USA), for which the upper limit of the reference interval was 388 pmol/L. Radioimmunoassay was performed on different aliquots of the same blood sample to those subsequently analyzed by LC–MS/MS. Biochemical data (creatinine, potassium) was obtained from a single reference laboratory (Idexx laboratories, Wetherby, UK) at the same time point.

PHA samples were obtained from cats seen in the above longitudinal study (*n* = 2) and cats seen in several different referral hospitals: Queen Mother Hospital for Animals (Royal Veterinary College, Hatfield, UK, *n* = 2), Cummings School of Veterinary Medicine (Tufts University, MA, USA, *n* = 1) and Pieper Veterinary (Pieper Veterinary, CT, USA, *n* = 1). Some cats were managed with surgical adrenalectomy, while others were managed medically. Biochemical data reported are from the closest available date to the collection of the sample used for LC–MS/MS but was not necessarily contemporaneous, and the biochemical results (including aldosterone quantification by RIA for three cats) were from local laboratories, so methodologies varied. PHA cases could receive potassium supplementation at the time of sampling. Methods of blood pressure measurement for cats in the PHA group from these referral hospitals were not standardized.

**FIGURE 1:**
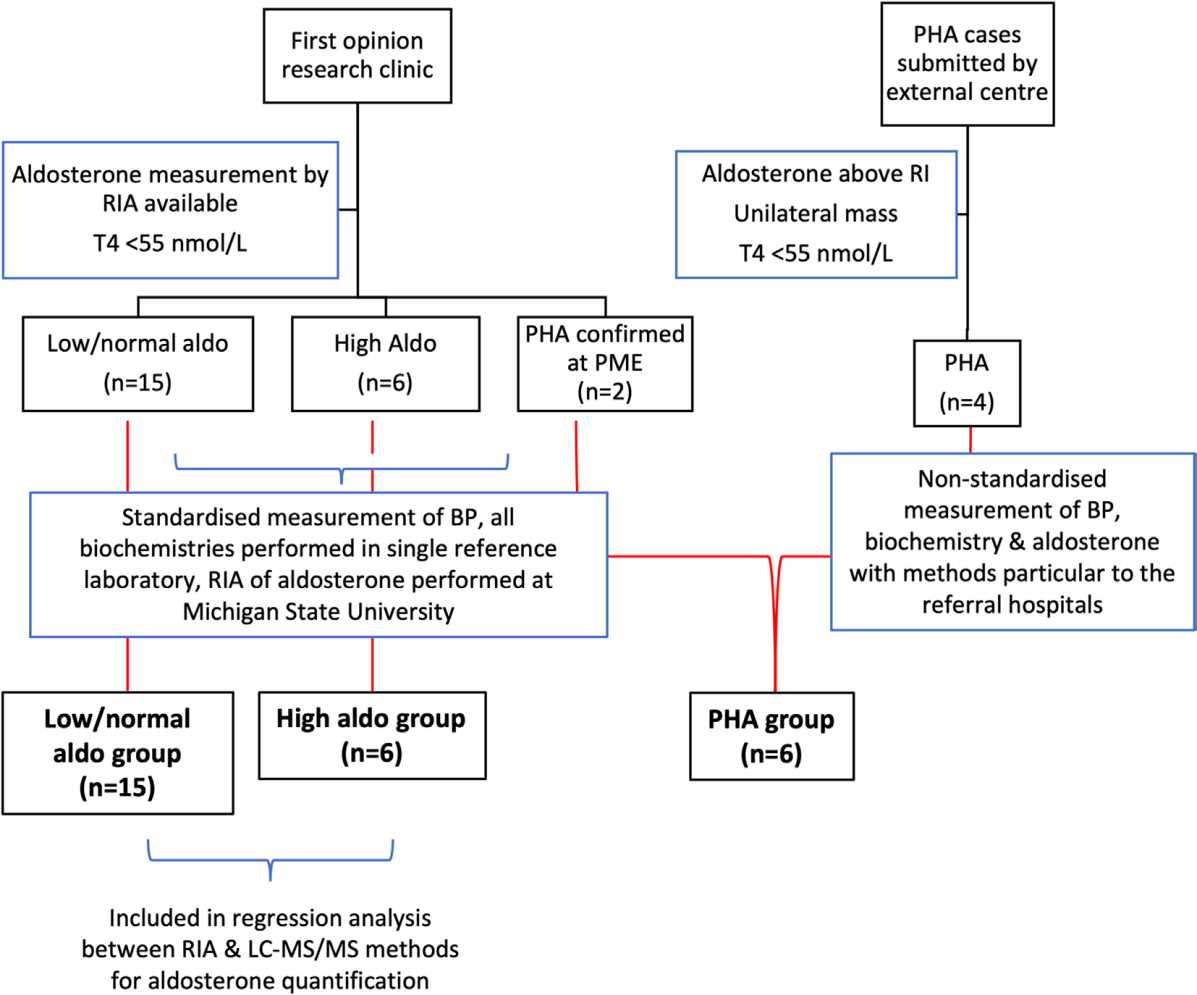
Flowchart depicting case inclusion criteria. RIA indicates radioimmunoassay; T4, total thyroxine; low/normal aldo, low or normal aldosterone measured by RIA; high aldo, high aldosterone measured by RIA; PHA, primary hyperaldosteronism; PME, post mortem examination; BP, blood pressure; and LC–MS/MS, liquid chromatography tandem mass spectrometry.

Residual serum and plasma samples were used in this study; samples were collected as indicated clinically by jugular venipuncture as described previously [16], and residual samples were stored at −80°C until analysis. The Royal Veterinary College's Ethics and Welfare Committee approved the collection and use of samples (URN 20131258E and URN 20212060‐3). Inclusion criteria for the three groups are summarized in Figure 1.

### 2.2 Steroid Analysis

Steroids were quantified using LC–MS/MS via a multi‐steroid profiling method for the identification and quantification of 25 steroids carried out on a Waters Xevo‐XS, with Acquity UPLC, using a water/methanol (0.1% formic acid) gradient system and a HSS T3, 1.8 μm, 1.2 × 50 mm column [17]. Briefly, after the addition of an internal standard and protein precipitation using 50 μL of acetonitrile, steroids were extracted from 200 μL of serum or plasma by liquid–liquid extraction using 1 mL of *tert*‐butyl methyl ether (MTBE). The MTBE layer containing steroids was transferred and evaporated under nitrogen and then reconstituted in 125 μL of 50/50 methanol/water prior to analysis. Quantification was against a calibration series ranging from 0.05 to 250 ng/mL. The lower limit of quantification (LLOQ) is reported for each steroid detected in the samples analyzed in this study (Table 1); the LLOQ was calculated on human plasma in a previous study [17]. Where steroid concentrations were below the LLOQ, an arbitrary value of LLOQ/2 was assigned. Comparison of LC–MS/MS and RIA quantification of aldosterone was performed on aliquots of plasma or serum from the same blood draw, with intervals of 6–24 months between analyses. PHA cases were excluded from this analysis due to a lack of contemporaneous samples. For the evaluation of the effect of sex on steroid profiles, a comparison between males and females was made using only samples obtained from cats with low/normal aldosterone concentration by RIA, to avoid any influence due to hyperaldosteronism.

**TABLE 1:** Comparing steroid concentrations in male and female neutered cats.

Steroid	LLOQ	FN		MN		*p*	Adj *p*
		(nmol/L)	*n*	(nmol/L)	*n*		
11‐deoxy‐corticosterone	0.45	< LLOQ (< LLOQ–1.59)	3/7	0.71 (< LLOQ–1.93)	7/8	0.18	0.45
Corticosterone	0.29	9.0 [3.6–34.1]	7/7	13.3 (2.7–23.1)	8/8	0.54	0.58
Aldosterone	0.42	< LLOQ (< LLOQ–0.48)	1/7	< LLOQ	0/8	0.35	0.57
Progesterone	0.32	1.1 (0.6–1.8)	7/7	1.0 (0.4–1.4)	8/8	0.54	0.58
11‐deoxycortisol	0.43	0.57 (< LLOQ–0.79)	4/7	0.92 (< LLOQ–1.16)	5/8	0.28	0.57
Cortisol	0.28	161 (76–241)	7/7	129 (23–231)	8/8	0.46	0.58
Cortisone	0.28	48 (31–76)	7/7	24 (20–39)	8/8	**0.0093**	0.083
Pregnenolone	0.63	8.4 (4.4–22.1)	7/7	6.1 (< LLOQ–8.8)	7/8	0.054	0.23
11‐keto‐androstenedione	0.33	0.59 (0.27–1.24)	6/7	0.28 (0.11–1.23)	3/8	0.12	0.39
11‐keto‐testosterone	0.33	< LLOQ (< LLOQ–0.52)	2/7	< LLOQ (< LLOQ–0.41)	1/8	0.51	0.58
11β‐hydroxy‐androstenedione	0.33	3.4 (0.4–5.0)	7/7	2.2 (0.5–5.7)	8/8	1.00	1.00
5α‐androstanedione	0.52	< LLOQ (< LLOQ–0.58)	1/7	< LLOQ	0/8	0.35	0.57
5α‐dihydro‐progesterone	0.63	2.8 (1.1–5.2)	7/7	0.7 (< LLOQ–2.6)	6/8	**0.013**	0.083

*Note:* Comparison of steroid concentrations measured by LC–MS/MS female neutered (FN, *n* = 7) and male neutered (MN, *n* = 8) cats from the low/normal aldosterone group. Measurements are summarized as median (range). Where steroids were below the lower limit of quantification (LLOQ) an arbitrary value of LLOQ/2 was assigned. Groups were compared using Mann Whitney *U* tests with Benjamini Hochberg correction. UD indicates below the limit of detection; *n*, number of samples above the LLOQ; and Adj *p*, adjusted *p*‐value. Bold values indicate statistically significant (*p* < 0.05).

### 2.3 Statistical Comparison of Aldosterone Quantification Methods

Statistical analyses were performed using R version 4.1.2 with statistical significance set at *p* < 0.05. Aldosterone quantification by RIA^1^ was compared with the multi‐steroid LC–MS/MS methodology using Pearson's correlation; PHA cases were excluded for this part of the study. Passing–Bablok and Bland–Altman analysis were then applied, and cases annotated as hypertensive or CKD were included. A multiple variable linear regression model was then performed, with aldosterone concentration measured by LC–MS/MS as the dependent variable and aldosterone measured by RIA and creatinine concentration as predictive variables.

### 2.4 Statistical Comparison Between Groups

Continuous numerical variables did not have a Gaussian distribution, so data is presented as median (range). Categorical data were compared using Fisher's exact test. The effect of sex on steroid profiles was investigated in cats without overt PHA, and steroids were compared using the Mann Whitney U tests using the Benjamini and Hochberg method to correct for multiple comparisons [18]. Comparison of steroid concentrations between the low aldo, high aldo, and PHA groups was performed using the Kruskal Wallis ANOVA with correction for multiple comparisons. Following a significant difference in the Kruskal–Wallis test, post hoc pairwise comparisons were conducted using Mann Whitney *U* tests. No power analysis was performed since no similar study has been performed on feline samples.

## 3 Results

### 3.1 Clinical Data

Cats in the study were all neutered, over 6 years old, and groups were balanced with respect to age, breed, and sex (Table 2). Many cats had azotemic chronic kidney disease in the low aldo (10/15), high aldo (6/6) and PHA groups (3/6). Fewer cats in the low aldo group were hypertensive. One cat in each of the high aldo and PHA groups was hyperthyroid. Potassium supplementation might have been administered to cats in the PHA group at the time of sampling. Cats in the PHA group were all hypokalemic before potassium supplementation, and 4/5 were documented to be hypertensive; blood pressure was not measured in one case. In three of the cats, the adrenal tumor tissue was collected at the time of surgical adrenalectomy, and in three cats, this was harvested during post‐mortem examination. In cats with PHA seen at the first opinion clinic, the presence of an adrenal mass was suspected ante‐mortem but was not confirmed by diagnostic imaging (*n* = 1) or was an incidental finding (*n* = 1). For the remaining cat with an adrenal tumor removed post‐mortem, the diagnosis of an aldosterone‐producing adrenal tumor had been made in life based on clinical presentation and diagnostic imaging (computed tomography and ultrasound) but the owner declined surgery and elected for medical management. PHA cases were managed with spironolactone (*n* = 3), amlodipine (*n* = 4) and potassium supplementation (*n* = 3), and concurrent conditions were managed with renal diet, meloxicam, furosemide, and thiamazole (*n* = 1 each). Five of the six adrenal masses were classified by board‐certified pathologists as adrenocortical carcinomas, with one of these cases also having distant lung metastases; the remaining case was classified as an adenoma. Where measurements were reported (*n* = 5) the largest dimension of the adrenal masses ranged from 1.5 to 10 cm. The PHA case with an adenoma had a lower aldosterone concentration (586 pmol/L) than the cats with ACCs but did not have the smallest adrenal tumor (2.3 cm). Aldosterone concentration measured by LC–MS/MS was different between groups (*p* < 0.001) and was lower in the high aldo group than the PHA group (*p* = 0.026).

**TABLE 2:** Descriptive statistics of cases.

		Low aldo (*n* = 15)	High aldo (*n* = 6)	PHA (*n* = 6)
Age (years)		15.0 (6.0–19.0)	16.7 (11.7–20.6)	13.7 (13.3–14.8)
Breed (Birman:DLH:DSH)		1:1:13	0:2:4	0:3:3
Sex (FN:MN)		7:8	4:2	3:3
Hypertension status (Y:N)		1:14	4:2	4:1^a^
Hyperthyroid status (Y:N)		0:15	1:5	1:5
Potassium (mEq/L)		3.90 (3.13–4.45)^b^	3.34 (3.07–3.70)	3.06 (2.42–3.94)^b^, ^c^
Creatinine	(μmol/L)	248 (178–1087)	192 (87–808)	151 (104–388)^c^
	(mg/dL)	2.8 (2.0–12.3)	2.2 (1.0–9.1)	1.7 (1.2–4.4)
Aldosterone^d^	(pmol/L)	147 (10–480)	683 (370–1030)	3061 (586–30 803)

*Note:* Descriptive statistics for high aldosterone measured by RIA (high aldo, *n* = 6), low/normal aldosterone measured by RIA (low aldo, *n* = 15) and primary hyperaldosteronism (PHA, *n* = 6) groups, giving median (range). High aldo indicates high aldosterone by RIA; low aldo, low/normal aldosterone by RIA; PHA, primary hyperaldosteronism; DLH, domestic longhair; DSH, domestic shorthair; FN, female neutered; MN, male neutered; Y, yes; and N, no.

^a^ BP not measured in one case.

^b^ Cats might have received potassium supplementation at the time of the sampling.

^c^ Not measured on the same sample or using the same methodology.

^d^ Aldosterone measured by mass spectrometry.

### 3.2 Comparing Aldosterone Quantification Methods

**FIGURE 2:**
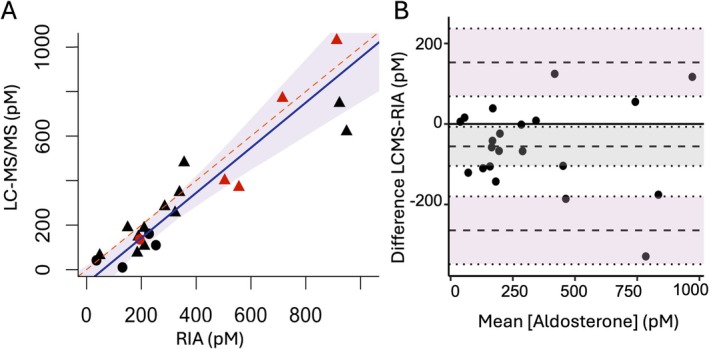
Comparison of aldosterone measurement by liquid chromatography tandem mass spectrometry (LC–MS/MS) and radioimmunoassay (RIA) for cats without overt primary hyperaldosteronism (*n* = 21). (A) Passing–Bablok analysis yielded the equation LC–MS/MS = 1.01 (RIA)–60.5, Pearson's linear correlation coefficient *r* = 0.93, *n* = 21. Blue line: fitted regression line, shading: 95% confidence interval, red dashed line: identity. Point color depicts hypertension status: red; hypertensive and black; normotensive. Point shape depicts CKD status: circles; non‐azotemic and triangles; azotemic chronic kidney disease. (B) Bland–Altman analysis demonstrated a mean negative bias of −56 pmol/L (95% CI −104 to −7) for the LC–MS/MS method. Dashed lines give upper limit of agreement (±1.96 SD), mean bias and lower limit of agreement and shading gives the 95% confidence intervals with dotted lines at the limits.

Paired aldosterone measurements performed on plasma aliquots by RIA and LC–MS/MS (*n* = 21) were significantly highly linearly related (*r* = 0.93, *p* < 0.001; Figure 2A), but values obtained by LC–MS/MS were consistently lower than those obtained by RIA (bias −56 pmol/L, 95% confidence interval −104 to −7; Figure 2B). The bias between measurement methods was relatively constant across a range of aldosterone values, and azotemic status did not alter this relationship. Multiple linear regression indicated that creatinine concentration (*p* = 0.95) did not alter the strength of the association between aldosterone concentrations measured by the two different methods (*R* ^2^ = 0.88, *p* < 0.001).

### 3.3 Steroid Profiles in Cats Without PHA

A mixture of serum (*n* = 19) and plasma (*n* = 8) samples was subjected to steroid measurement by LC–MS/MS. Of the 25 steroids investigated, 12 steroids were below the LLOQ including androstenedione, dehydroepiandrosterone (DHEA), androstanediol, 5α‐androsterone, allo‐pregnanolone, 17‐hydroxy‐hallopregnanolone, 17‐hydroxypregnenolone, 17‐hydroxyprogesterone, testosterone, 5α‐dihydrotestosterone, 11‐hydroxytestosterone, and 17α‐hydroxy‐5α‐dihydroprogesterone. Thirteen steroids were detectable in one or more cats across all groups (Table 1). Female (*n* = 7) and male (*n* = 8) neutered cats with aldosterone concentrations below the upper limit of the reference range had similar ages (median 15.1 and 15 years, respectively) and creatinine concentrations (median 240 and 152 μmol/L or 2.71 and 1.72 mg/dL, respectively). None of the measured glucocorticoids were present at different concentrations between the sexes after correcting for multiple comparisons; although, without correction for multiple comparisons, cortisone was higher (*p* = 0.0093, adjusted *p* = 0.083) in females (48, range 31–76 nmol/L) than males (24, range 20–39 nmol/L). Otherwise, no differences in steroid concentrations were detected between male and female blood steroid profiles. 11β‐hydroxyandrostenedione was the 11‐oxygenated androgen detected at the highest concentration in neutered cats.

### 3.4 Comparison of Steroid Profiles Between Groups

**FIGURE 3:**
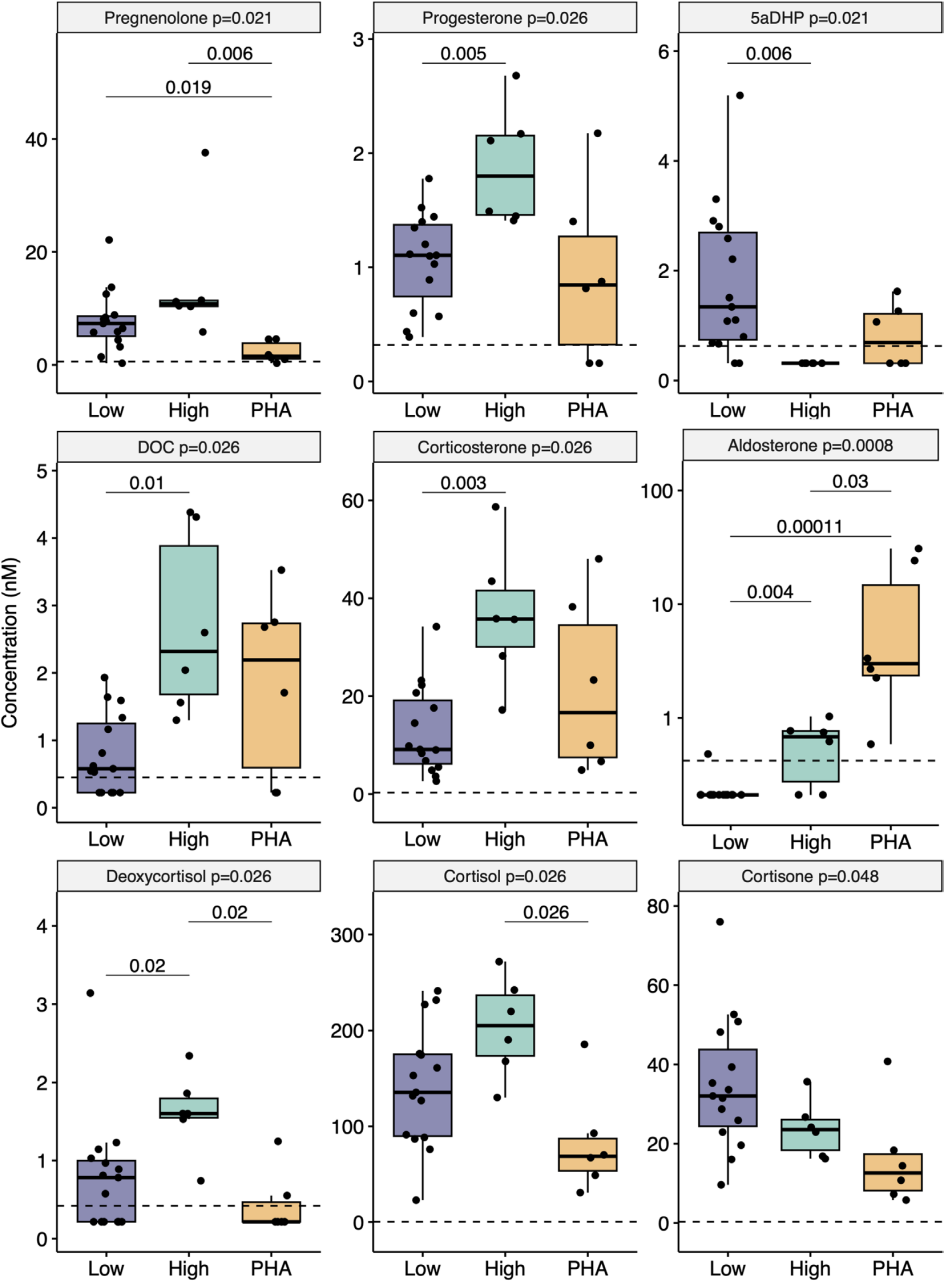
Boxplots with overlying circles representing each data point for steroids with significantly different concentrations between the three groups. Low/normal aldosterone by RIA in purple (low, *n* = 15), high aldosterone by RIA in green (high, *n* = 6), and primary hyperaldosteronism in yellow (PHA, *n* = 6). *Y*‐axes give steroid concentration in nmol/L on a linear scale except for aldosterone which is logarithmic. The lower limit of quantification (LLOQ) depicted as a horizontal dashed line, all values below the LLOQ were assigned a value of LLOQ/2. Each row of graphs represents common precursors, mineralocorticoids and glucocorticoids respectively. *p*‐values on panel label are from Kruskal–Wallis one way ANOVA, *p*‐values as annotations give post hoc comparisons with Benjamini Hochberg correction. Boxes depict the 25th, 50th and 75th percentiles; whiskers extend from the hinge to the largest value not more than 1.5× the interquartile range from the hinge. DOC indicates 11‐deoxycorticosterone; deoxycortisol, 11‐deoxycotisol; and 5aDHP, 5α‐dihydroprogesterone.

In addition to aldosterone, 10 steroids were significantly different between cats with low aldo (*n* = 15), high aldo (*n* = 6) and PHA (*n* = 6; Figure 3). These included four precursor steroids (pregnenolone, progesterone, 17‐hydroxyprogesterone and 5α‐dihydroprogesterone), two mineralocorticoids (deoxycorticosterone and corticosterone), three glucocorticoids (11‐deoxycortisol, cortisol and cortisone) and one androgen (androsterone). Although statistically different between groups, 17‐hydroxyprogesterone and androsterone were only above LLOQ in a minority of the cats, and so these results are relegated to Figure S1.

Compared with the low aldo group, the high aldo group had significantly higher concentrations of the common steroid precursors (progesterone and 17‐hydroxyprogesterone), mineralocorticoids (deoxycorticosterone, corticosterone) and 11‐deoxycortisol and significantly lower concentrations of 5α‐dihydroprogesterone. Androsterone was the only steroid with a significantly higher concentration in the PHA group than the low aldo group (*p* = 0.04). Compared with the high aldo group, the PHA group had significantly lower concentrations of the common steroid precursors pregnenolone and progesterone and glucocorticoids (including 11‐deoxycortisol and cortisol).

## 4 Discussion

Aldosterone measurements obtained by RIA and LC–MS/MS in this study were shown to be strongly related; although quantification by LC–MS/MS was lower, as expected, since this has also been seen with human samples [19]. Plasma creatinine concentration was not associated with discrepancies between RIA and LC–MS/MS methods for aldosterone quantification, indicating that aldosterone quantification can be performed using either RIA or LC–MS/MS in clinical practice for the diagnosis of PHA. Since absolute measurements were different by the two methods, method‐specific reference intervals must be used and remain to be established for this LC–MS/MS method. This study also identified differences in multi‐steroid profiles on 200 μL of cat serum or plasma from cohorts of cats with low aldo, high aldo, and PHA.

Aldosterone concentration can be spuriously elevated in humans with kidney disease when measurements are made by RIA [11]. This falsely high aldosterone concentration arises due to the accumulation of polar metabolites, which can be removed by solvent extraction before RIA to provide reliable results [9, 10]. In the present study, we found no evidence for polar metabolites interfering in the RIA because the relationship between aldosterone measurements by RIA and LC–MS/MS (which would not be affected by polar metabolites) was not affected by azotemia. This finding could be in accordance with the observation of minimal aldosterone metabolites in previous studies of feline urine [20], indicating that aldosterone metabolites produced in cats might be different from those formed in humans, possibly due to differences in downstream metabolism or conjugation [21]. It should be noted that most cats in the low and high aldo groups had elevated creatinine concentrations, as cases were selected from a cohort of cats with CKD that had endocrine testing performed for a previous study.

In humans, there are sex‐related differences in steroid hormone concentrations [22]. Steroid profiles were not different between male and female neutered cats in this study. Due to the small sample size, the power was somewhat limited, and it is possible that subtle differences between the sexes could have been missed, but the lack of difference could also reflect the influence of neutering. Androgen concentrations were low in all neutered cats, indicating a lack of adrenal steroidogenesis, similar to rats, mice, and ferrets [23, 24]. Of note, androstenedione and DHEA were undetectable (LLOQ 0.7 and 0.69 nmol/L, respectively). In a previous study of 3‐year‐old neutered cats, androstenedione and DHEA concentrations were 0.24–1.92 nmol/L by immunoassay and 0.31–1.63 nmol/L by mass spectrometry, respectively [8]. A similar androstenedione reference interval is given in another study [25]. The present study looked at older cats, and the production of these steroids might reduce with age; alternatively, residual androgens produced by gonads could have been present in the younger cats as the date of neutering was not reported. DHEA is undetectable in species other than humans and primates as DHEA and DHEA sulfate are only produced in the adrenal cortex of these species [26, 27].

11β‐hydroxyandrostenedione was the most abundant 11‐oxygenated androgen in feline blood, which is similar to other species, including pigs, guinea pigs, and primates [28]. In humans, 11β‐hydroxyandrostenedione is produced from androstenedione, catalyzed by the enzyme CYP11B1 [29], and to a lesser degree from cortisol [30]. Since neutered cats had minimal circulating androstenedione concentrations, it is likely that the 11β‐hydroxyandrostenedione was being produced from cortisol. Transfection experiments of genes encoding relevant enzymes (CYP11B and CYP17A1) and treatment with steroids, followed by measurement of steroids in culture medium, could help to clarify the major routes of steroid synthesis in cats.

Median concentrations of deoxycorticosterone measured in cats in this study were higher than the upper 97.5 percentile for human blood deoxycorticosterone concentrations (0.49 nmol/L) [31], suggesting differences in steroid metabolism in cats. This is supported by a previous study where deoxycorticosterone was quantified by immunoassay [8]. This could be a result of differing kinetics of the feline multifunctional CYP11B enzyme compared to the separate cortisol and aldosterone synthase enzymes found in humans.

The multi‐steroid profile revealed differences between steroid concentrations in three groups: PHA, low aldo, and high aldo. Of note, the PHA group had lower glucocorticoid concentrations (including cortisol) than the low aldo group, while the high aldo group had higher glucocorticoid production (excluding cortisol) than the low aldo group. This contrasts with PHA in humans, where some adrenal tumors secrete cortisol alongside aldosterone [32]. It is possible that the acute stress of veterinary visits could stimulate ACTH‐dependent glucocorticoid production in cats, which might be absent in the PHA cases if tumors are autonomously secreting hormones that suppress ACTH secretion, or the effect might be blunted by the large amount of steroid already in circulation. Dynamic ACTH stimulation tests could be performed to investigate the adrenal reserve for cortisol production in PHA cases. Many of the cats in the low and high aldo groups had relatively high creatinine concentrations. This was due to the enrichment of the study population for cats with relatively severe azotaemia, so that we could address the clinical question of whether RIA performance would be altered in this situation. In contrast, most of the PHA group were non‐azotaemic. As would be anticipated, there were more hypertensive cats in the high aldo and PHA groups than in the low aldo group, and this could be attributed to aldosterone excess causing hypertension.

Excess production of multiple steroids has been reported in cats with hyperaldosteronism [15], this was not evident in the cohort of cats with PHA reported here, although the number of cats investigated was very small. 11/12 cats with PHA reported in the literature with concurrent progesterone hypersecretion were diabetic [12, 33, 34]. None of the included PHA cases had concurrent diabetes. Pregnenolone, a common precursor of many steroids analyzed, had concentrations that were significantly lower in the PHA group than in either high or low aldo groups.

The guidelines for PHA diagnosis in humans recommend PRA measurement [1], however, a limitation of our work was that PRA was not measured for all cats with PHA. The classification system used to define PHA cases in this study (unilateral tumor with aldosterone excess) has been used in other feline studies due to a lack of availability of PRA assays [35], however, this classification system likely only selects for the extreme cases of PHA with large tumors and severe clinical signs. Cats in the high aldo group could have primary or secondary hyperaldosteronism; however, no information about adrenal size was available. It is therefore interesting that the pattern of changes was different in the high aldo and PHA groups, suggesting that the pathogenesis of excess aldosterone might be different in the high aldo and PHA groups.

Available literature on feline PHA cases with a histopathological diagnosis reports 59% (58/99) as adrenocortical carcinomas (ACC) and 37% (37/99) as adenomas [3, 12, 13, 14, 33, 34, 36, 37, 38, 39, 40, 41, 42, 43, 44, 45, 46, 47, 48, 49]. In contrast, most aldosterone‐secreting tumors in humans are benign adenomas, and only around 2.5% are adenocarcinomas [50]. This could be because of higher screening rates in humans, with many adrenal masses being detected incidentally. For cats, routine imaging is less common; small adenomas can be challenging to detect, and mild signs mean PHA might remain underdiagnosed [2], with only cases with severe aldosterone excess being detected. Longer and more detailed follow up of humans after removal of aldosterone‐producing adrenal tumors means that metastasis or recurrence of tumors is closely monitored.

Multi‐steroid profiling has emerged as a valuable diagnostic tool in humans. It aids in differentiating adrenocortical adenomas from carcinomas with high specificity and can identify patterns associated with prognosis [51, 52]. Such feline specific classification systems to differentiate benign and malignant adrenal tumors do not exist. Within this PHA cohort, five out of six cases were classified as ACCs, and whilst three of these showed malignant behavior (macroscopic invasion of the caudal vena cava or distant metastasis), it is possible that the remaining tumors were misclassified. ACCs in humans tend to secrete precursor steroids including 11‐deoxycortisol, 11‐deoxycorticosterone, and 17‐hydroxyprogesterone, or their urinary metabolites tetrahydro‐11deoxycortisol, 5‐pregnenediol, and 5‐preganen‐triol [51, 52, 53, 54]. Precursor steroid excesses were not observed in this feline PHA cohort; 11‐deoxycortisol and 17‐hydroxyprogesterone were significantly lower in the PHA group.

Humans with unilateral PHA have higher serum concentrations of hybrid steroids (18‐hydroxycortisol and 18‐oxocortisol) than patients without PHA [55], and this is particularly common in patients with tumors containing KCNJ5 mutations [56, 57, 58]. 18oxo‐cortisol and 18‐hydroxycortisol were not included in this assay, but it would be interesting to investigate the concentrations of these steroids in feline blood, especially within a cohort of cats with PHA. Many factors including age and body mass index have been reported to affect blood steroid profiles in humans [31], it was not possible to control for all potentially implicated factors within this cohort of cats.

Due to the retrospective nature of this study, both serum and plasma samples were included in the analysis. Serum samples were preferred, as serum has lower matrix effects than plasma. To overcome this issue as much as possible, internal standards were incorporated, which should account for some of the matrix effects. This is a limitation of the study, and future research will focus on either serum or plasma only. Diagnostic imaging of the abdomen is not routinely performed on cats evaluated in the first opinion clinic (all cats in the high and low aldo groups and two PHA cats), and for some cases, aldosterone measurements were performed on archived blood samples after the cat had died, precluding further diagnostic evaluation. Furthermore, this study did not include a healthy control group.

This study suggests that both LC–MS/MS and RIA can be used for the quantification of aldosterone, but assay‐specific reference intervals should be used when interpreting the results. Azotemia was not observed to affect the performance of RIA quantification of aldosterone, suggesting that RIA measurement remains appropriate for cats with CKD. The multi‐steroid profile was used to compare steroid production between different groups of cats, including those with PHA, and might be applicable to other steroid endocrinopathies, including hypercortisolism or hypoadrenocorticism. Steroid profiling has also been used to identify steroid enzymopathies such as in congenital hyperplasia in humans [59], and could have similar applications for veterinary patients with a single test rather than requiring large volumes of blood for multiple immunoassays.

## Disclosure

Authors declare no off‐label use of antimicrobials.

## Ethics Statement

Royal Veterinary College's Ethics and Welfare Committee approved the collection and use of samples (URN 20131258E and URN 20212060‐3). Authors declare that human ethics approval was not needed.

## Conflicts of Interest

The authors declare no conflicts of interest.

## Supporting information


**Figure S1:** Boxplots with overlying circles representing each data point for steroids without significantly different concentrations between the three groups. Low/normal aldosterone by RIA in purple (low, *n* = 15), high aldosterone by RIA in green (high, *n* = 6), yellow and primary hyperaldosteronism in yellow (PHA, *n* = 6). *Y*‐axes give steroid concentration in nmol/L on a linear scale with the lower limit of quantification (LLOQ) depicted as a horizontal dashed line. *p*‐values on panel labels are from Kruskal–Wallis one way ANOVA, *p*‐values annotated on graphs give post hoc comparisons with Benjamini Hochberg correction. Boxes depict the 25th, 50th and 75th percentiles; whiskers extend from the hinge to the largest value not more than 1.5× the interquartile range from the hinge. Values below the lower limit of quantification (LLOQ) were allocated values of LLOQ/2.17OHP indicates, 17‐hydroxyprogesterone; 11KA4,11‐ketoandrostenedione; 11KT, 11‐ketotestosterone; 11OHA4, 11‐hydroxyandrostenedione; and 11OHT, 11‐hydroxytestosterone.

## Endnotes

^1^
Michigan State University Veterinary Diagnostic Laboratory, USA.
