# Supplementary material for: Determination of Multi‐Steroid Profiles of Cats With Hyperaldosteronism or Other Diseases: A Retrospective Study
Source: J Vet Intern Med. 2025 Aug 14;39(5):e70209. doi: 10.1111/jvim.70209 (PMC12353250; doi:10.1111/jvim.70209)
Supplement: Supplementary file 1 — Figure S1: Boxplots with overlying circles representing each data point for steroids without significantly different concentrations between the three groups. Low/normal aldosterone by RIA in purple (low, n = 15), high aldosterone by RIA in green (high, n = 6), yellow and primary hyperaldosteronism in yellow (PHA, n = 6). Y‐axes give steroid concentration in nmol/L on a linear scale with the lower limit of quantification (LLOQ) depicted as a horizontal dashed line. p‐values on panel labels are from Kruskal–Wallis one way ANOVA, p‐values annotated on graphs give post hoc comparisons with Benjamini Hochberg correction. Boxes depict the 25th, 50th and 75th percentiles; whiskers extend from the hinge to the largest value not more than 1.5× the interquartile range from the hinge. Values below the lower limit of quantification (LLOQ) were allocated values of LLOQ/2.17OHP indicates, 17‐hydroxyprogesterone; 11KA4,11‐ketoandrostenedione; 11KT, 11‐ketotestosterone; 11OHA4, 11‐hydroxyandrostenedione; and 11OHT, 11‐hydroxytestosterone. [file JVIM-39-e70209-s001.docx]

**Supplementary Figure 1**

Boxplots with overlying circles representing each data point for steroids without significantly different concentrations between the three groups. Low/normal aldosterone by RIA in purple (low, n=15), high aldosterone by RIA in green (high, n=6), yellow and primary hyperaldosteronism in yellow (PHA, n=6). Y-axes give steroid concentration in nmol/L on a linear scale with the lower limit of quantification (LLOQ) depicted as a horizontal dashed line. P-values on panel labels are from Kruskal Wallis one way ANOVA, P-values annotated on graphs give post hoc comparisons with Benjamini Hochberg correction. Boxes depict the 25^th^, 50^th^ and 75^th^ percentiles; whiskers extend from the hinge to the largest value not more than 1.5 x the interquartile range from the hinge. Values below the lower limit of quantification (LLOQ) were allocated values of LLOQ/2. 17OHP indicates, 17-hydroxyprogesterone; 11KA4,11-ketoandrostenedione; 11KT, 11-ketotestosterone; 11OHA4, 11-hydroxyandrostenedione; and 11OHT, 11-hydroxytestosterone.
